# Evaluating the relationship between blood lipids, cardiac risk index, and suicidal behaviors in first-episode psychosis

**DOI:** 10.1192/j.eurpsy.2024.605

**Published:** 2024-08-27

**Authors:** L. Güçlü, M. Şen, Y. H. Balcıoğlu, P. Çelikkıran, S. S. Kırlıoğlu Balcıoğlu, H. Gökçay, N. Karamustafalıoğlu, Ü. H. Yeşilkaya

**Affiliations:** ^1^Faculty of Medicine, Bahcesehir University; ^2^Psychiatry, Bakirkoy Prof. Mazhar Osman Training and Research Hospital for Psychiatry- Neurology- and Neurosurgery; ^3^Psychiatry, Basaksehir Cam and Sakura City Hospital; ^4^Psychiatry, Bagcilar Training and Research Hospital, Istanbul, Türkiye

## Abstract

**Introduction:**

Schizophrenia patients have demonstrated a high prevalence of suicidal behavior. There isn’t yet a definitive explanation of the neurobiological mechanisms of suicidal behavior (SB) in psychotic patients. While subthreshold indices of dyslipidemia were observed in schizophrenia patients, it has shown that there is a relationship between dyslipidemia and suicidal behavior.

**Objectives:**

The study aimed to investigate possible associations between SB and lipid profile including cardiovascular biomarkers in first-episode psychosis (FEP) as compared with healthy controls (HC).

**Methods:**

The study sample consisted of 173 subjects (111 male, 62 female). The sample included 31 drug- naïve FEP patients with current SB (FEP+S), 66 drug-naïve FEP patients without SB (FEP-S), and 76 mentally and medically HC subjects. Blood samples were collected from all participants to determine total cholesterol (TC), low- and high-density lipoproteins (LDL and HDL), and triglycerides (TG). Castelli Risk Index-I, Castelli Risk Index-II, and atherogenic index were calculated. Symptoms were assessed by using the Positive and Negative Syndrome Scale. Nonparametric Kruskal-Wallis and Mann-Whitney tests were used for pairwise comparisons of account of lipid parameters in three groups. A binomial logistic regression analysis was performed to examine the predictive power of lipid profile in the presence of SB in FEP.

**Results:**

The results of Kruskal-Wallis test revealed that a statistical difference was found in TC and TG between groups. Despite statistical significance was observed in TG between all patient groups (FEP+S and FEP-S) and controls, as well as in TC between FEP-S and controls, there was no significant difference between FEP-S and FEP+S for any lipid parameters or cardiovascular biomarkers. No significant correlation was identified between lipid profile and symptom severity. No significant relationships were able to predict the presence of SB in FEP.

**Conclusions:**

In this study, plasma levels of lipid parameters and biomarkers of cardiovascular risk in all patient groups (FEP+S and FEP-S) and HC were compared. Current results show no linkage between SB and lipid profile, but significantly lower levels of cholesterol in FEP. Therefore, cholesterol might have a protective role in terms of psychosis while CI1, CI2, and AI were not increased in psychosis. Thus, increased cholesterol levels or cardiovascular risk in schizophrenia patients, that several studies found, may ensue after antipsychotic use. Overall, present findings suggest that lipid profile abnormalities are specifically associated with FEP rather than SB. Controversy of results may reveal that research on SB requires an understanding of whether the patient had concomitant psychosis.

**Disclosure of Interest:**

None Declared

